# Insulin Sensitivity and Muscle Loss in the Absence of Diabetes Mellitus: Findings from a Longitudinal Community-Based Cohort Study

**DOI:** 10.3390/jcm14041270

**Published:** 2025-02-14

**Authors:** Hyun Jung Kim

**Affiliations:** Department of Physical Medicine and Rehabilitation, Soonchunhyang University Bucheon Hospital, Soonchunhyang University College of Medicine, Bucheon-si 14584, Gyeonggi-do, Republic of Korea; dbyj01@gmail.com

**Keywords:** muscle loss, insulin sensitivity, mortality

## Abstract

**Background/Objectives**: Muscle loss is a serious complication in chronic disease patients, yet studies on long-term changes in muscle mass based on insulin sensitivity in the absence of diabetes mellitus are scarce. This community-based cohort study analyzed the longitudinal association between insulin sensitivity and muscle loss in middle-aged South Korean adults. **Methods**: This study included 6016 subjects (aged 40–65 years) from the Korean Genome and Epidemiology Study, conducted between 2001 and 2016. Fat-free mass, fat mass, body weight, and kidney function were assessed biennially. Subjects were categorized into four groups based on the composite (Matsuda) insulin sensitivity index (ISI) quartiles. The primary outcome was muscle loss, defined as a decline in fat-free mass of 10% or more from baseline. The secondary outcome was the occurrence of all-cause mortality. **Results**: During 69,480 person–years of follow-up, muscle loss occurred in 311 (5.2%) subjects. Multivariable Cox regression revealed a reverse-graded association between insulin sensitivity and muscle loss risk. Hazard ratios (95% confidence intervals) for the second, third, and highest ISI quartiles were 0.70 (0.51–0.94), 0.69 (0.50–0.95), and 0.65 (0.46–0.92), respectively, compared with the lowest quartile. Insulin sensitivity, however, was not significantly associated with all-cause mortality, though the mortality risk was higher in individuals with muscle loss. **Conclusions**: A reverse-graded relationship between insulin sensitivity and muscle loss risk was identified in middle-aged South Korean adults, with the lowest risk in the highest ISI quartile. These findings suggest that higher insulin sensitivity may reduce the risk of muscle loss.

## 1. Introduction

Skeletal muscle, which constitutes 40–50% of total body mass in individuals with a healthy weight, is not only essential for locomotion but also serves as a critical protein reservoir, playing a key role in maintaining the homeostasis of glucose, amino acids, and lipids [1]. Muscle loss occurs as a natural part of aging [2,3,4] and is further exacerbated by acute or chronic illnesses [5,6,7,8,9,10,11]. Previous studies have shown that patients with diabetes mellitus (DM) experience a more rapid decline in muscle mass compared to those without DM [9,11,12]. Substantial muscle loss is associated with poor prognoses in various conditions, including cancer, organ failure, infections, and age-related functional decline [13,14,15]. Moreover, reduced muscle mass has been associated with a heightened risk of mortality [16].

Insulin is a potent anabolic hormone that stimulates peripheral glucose uptake, suppresses hepatic glucose production, and prevents muscle proteolysis. It also promotes muscle protein synthesis and reduces lipolysis in adipose tissue [17]. Insulin sensitivity reflects the body’s ability to respond to insulin, particularly in enhancing glucose uptake, facilitating glucose disposal in peripheral tissues, and inhibiting hepatic glucose production. Increased insulin sensitivity allows for effective blood glucose regulation with lower insulin levels. Conversely, decreased insulin sensitivity (insulin resistance) impairs insulin’s action on muscle and fat, potentially resulting in the loss of lean and fat mass [12].

As individuals progress from normal glucose tolerance to impaired glucose tolerance, insulin sensitivity declines significantly, while the deterioration of glucose tolerance remains relatively mild due to a compensatory increase in insulin secretion [18]. Even in the presence of severe insulin resistance, fully functional β-cells can produce sufficient insulin to counterbalance impaired insulin action [19]. Thus, the development of type 2 DM requires abnormalities in both insulin secretion and insulin action. Importantly, individuals with severe insulin resistance do not necessarily develop type 2 DM, highlighting the complexity of the condition. Therefore, understanding the impact of insulin sensitivity on body composition in the general population without DM is critical.

However, to the best of my knowledge, no longitudinal study has examined the relationship between muscle loss and insulin sensitivity in individuals without a history of DM. In this study, the relationship between insulin sensitivity and muscle loss in middle-aged South Korean adults without a history of DM was investigated.

## 2. Materials and Methods

### 2.1. Ethical Guidelines Statement

All subjects voluntarily took part in this study and provided informed consent. The study protocol received an approval from the Ethics Committee of the Korean Genome and Epidemiology Study (KoGES) [20] at the Korean National Institute of Health. The study was conducted in accordance with the Declaration of Helsinki and obtained approval from the institutional review board (2021-02-018).

### 2.2. Study Subjects

**Figure 1:**
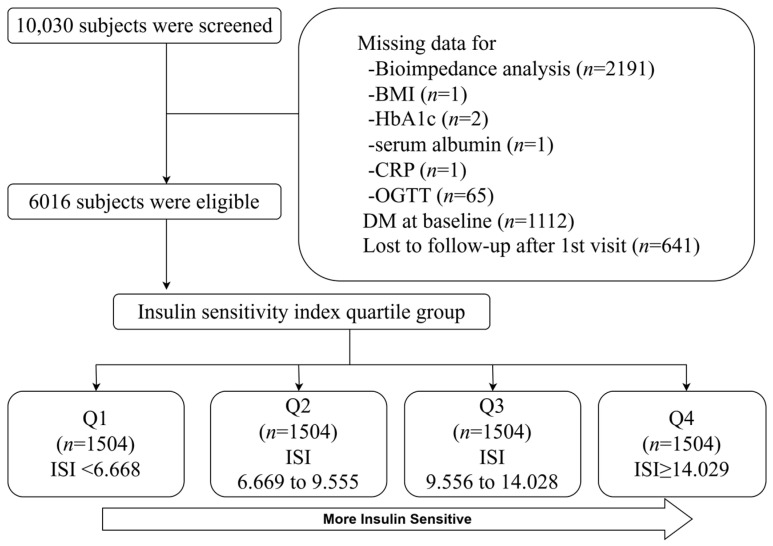
Flow diagram of study cohort.

A prospective community-based cohort study, known as KoGES, was used. The rationale, design, methods, and protocol summary have been previously described [20]. Briefly, the study cohort consisted of 10,030 subjects aged 40–69 years, all with a homogeneous ethnic background, recruited from the national health examinee registry. They were residents of Ansan (an urban area) and Ansung (a rural area), located near Seoul, Republic of Korea. In 2001, the subjects underwent anthropometric measurements, laboratory testing, and completed health-related lifestyle questionnaires at baseline, with the process repeated biennially until 2016. The retention rate was 71.4% by the end of the seventh follow-up phase. Key exclusion criteria included the following: (1) subjects who did not undergo bioimpedance analysis (BIA) at baseline (*n* = 2191); (2) those with DM at baseline (*n* = 1112); and (3) those lost to follow-up after the first visit (*n* = 641). Subjects with missing data and those lost to follow-up after the baseline visit were also excluded. A total of 6016 subjects were included in the final analysis (Figure 1). In this study, 6016 subjects were ranked by insulin sensitivity, from the lowest to the highest, and divided into four quartiles based on their insulin sensitivity levels. The first quartile (Q1) included subjects with an insulin sensitivity index (ISI) below 6.668, the second quartile (Q2) ranged from 6.669 to 9.555, the third quartile (Q3) from 9.556 to 14.028, and the fourth quartile (Q4) consisted of subjects with an ISI of 14.029 or higher.

### 2.3. Demographic, Anthropometric, and Laboratory Data

According to the site visit schedule, subjects underwent health exams that included anthropometric measurements, biological specimen collection, and interviews. Subjects were asked questions about demographics and socioeconomic status, including age, sex, income, education level, alcohol consumption, smoking status, and medical history, all administered by an interviewer. The validated food frequency questionnaire (FFQ), combined with the 24 h recall method, was used to assess the participants’ nutritional status. Height and weight measurements were taken while subjects were barefoot and lightly clothed, respectively. Height was recorded to the nearest 0.1 cm using a stadiometer, while weight was measured on a scale. Body mass index (BMI) was then calculated by dividing weight (kg) by height squared (m^2^). Physical activity levels were assessed as daily estimated metabolic equivalents of task (MET), categorized into quartiles based on a semi-quantitative survey questionnaire. One MET is equivalent to the oxygen consumption of 3.5 mL per kilogram of body weight per minute at rest. To calculate MET, subjects reported the number of hours they slept and the hours spent on five different activity levels: inactive, very light, light, moderate, and heavy. Total MET hours per day were calculated by multiplying the reported hours spent in each activity by the corresponding MET value for that activity level [21]. Education levels were categorized into three groups based on years of education: 6 years, 6–9 years, and >9 years. Monthly income was divided into three groups: $850, $850–$1699, and $1700 per month.

Urine samples were collected after the initial morning void, and a dipstick test was performed using the URISCAN Pro II (YD Diagnostics Corp., Seoul, Republic of Korea). Urine albumin levels were categorized on a color scale as absent, trace, 1+, 2+, or 3+. Albuminuria was defined as a dipstick test result of 1+ albumin.

Following an 8 h fast, blood samples were collected, transported to Seoul Clinical Laboratories (Seoul, Republic of Korea) within 24 h, and analyzed for serum blood urea nitrogen, creatinine, albumin, C-reactive protein (CRP), calcium, total cholesterol, high-density lipoprotein cholesterol (HDL-C), triglycerides, fasting glucose, hemoglobin, and glycated hemoglobin (HbA1c). During the follow-up, serum creatinine was measured using the Jaffe method, and non-isotope dilution–mass spectrometry (IDMS) creatinine values were converted to IDMS creatinine using the equation previously suggested [22,23]. The Chronic Kidney Disease Epidemiology Collaboration equation was then used to calculate the estimated glomerular filtration rate (eGFR) [24].

Insulin sensitivity was measured using the composite (Matsuda) ISI [25] and the homeostasis model assessment of insulin resistance index (HOMA-IR) [26]. Pancreatic β-cell function was estimated using the 60 min insulinogenic index (IGI_60_), calculated with plasma insulin and glucose levels at 0 and 60 min of during the oral glucose tolerance test (OGTT) [27].

### 2.4. Assessment of Body Composition

Multifrequency bioelectrical impedance analysis (BIA) was used to determine body composition (InBody 3.0, Biospace, Seoul, Republic of Korea). BIA was performed at the start of the study and every two years thereafter. Multifrequency BIA operates under the assumption that the human body consists of five interconnected cylinders, measuring impedance directly in these compartments, unlike traditional BIA approaches, which use equations to estimate the mass of each bodily component. A tetrapolar 8-point tactile electrode system was employed to assess impedance in five segments (both arms, trunk, and both legs) at four different frequencies (5, 50, 250, and 500 kHz).

### 2.5. Exposure and Outcome

The primary exposure of interest was the ISI, with subjects categorized into four groups based on ISI quartiles. The primary endpoint was the development of de novo muscle loss, defined as a 10% or greater decline in FFM from baseline, as measured by BIA.

Secondary outcomes included all-cause mortality. Primary outcome events were identified based on two or more event measurements, with the first occurrence designated as the study endpoint.

### 2.6. Statistical Analysis

Data analysis was conducted using R 4.1.1 (http://www.R-project.org, accessed on 2 September 2021). Continuous variables were expressed as means with standard deviations, while categorical variables were presented as absolute numbers and percentages. Prior to statistical analysis, all data were tested for normality using a Kolmogorov–Smirnov test. For continuous variables with a normal distribution, an analysis of variance (ANOVA) was employed, whereas categorical variables were analyzed using a Chi-square test or Fisher’s exact test. For non-normally distributed data, a Mann–Whitney U test or a Kruskal–Wallis test was applied. A Kaplan–Meier curve analysis [28] was used to estimate survival rates for study outcomes, and differences between groups were assessed with the log-rank test.

Survival time was defined as the interval between baseline and either the onset of the outcome or the last follow-up. Multivariable Cox proportional hazards regression models were constructed to examine the association of ISI with the risk of incident muscle loss and all-cause mortality. Variables that were statistically significant in univariable analysis were included in the multivariable models. Although the *p*-values for certain factors were above 0.05, clinically important variables such as cancer history, COPD history, HDL-C, CRP, and albuminuria were included in the analysis due to their potential clinical relevance. Model 1 was unadjusted. Model 2 was adjusted for baseline age, sex, BMI, systolic blood pressure (SBP), HbA1c, eGFR, albuminuria, HDL-C, serum calcium, serum albumin, and CRP. Model 3 included additional adjustments for demographic factors (education level, economic status, alcohol use, smoking status, and physical activity measured as MET) and comorbidities, such as cardiovascular disease (myocardial infarction, congestive heart failure, unstable angina, peripheral artery disease, and cerebrovascular disease), previous cancer, and chronic obstructive pulmonary disease. *p*-values < 0.05 were considered statistically significant.

## 3. Results

### 3.1. Baseline Characteristics

The baseline characteristics of the study population are summarized in Table 1. The mean age of the subjects was 50.9 ± 8.6 years, and 47.4% were male. The average eGFR was 91.9 ± 13.8 mL/min/1.73 m^2^. SBP was significantly higher in subjects in the lowest quartile, who were also more likely to have hypertension. Additionally, BMI, lean soft mass (LSM), FFM, fat-free mass index (FFMI), fat mass (FM), and fat mass index (FMI) were higher in the lowest quartile compared to the higher quartiles. No significant dietary differences were observed between the groups (Supplemental Table S1).

### 3.2. Association Between Insulin Sensitivity and Incident Muscle Loss

**Figure 2:**
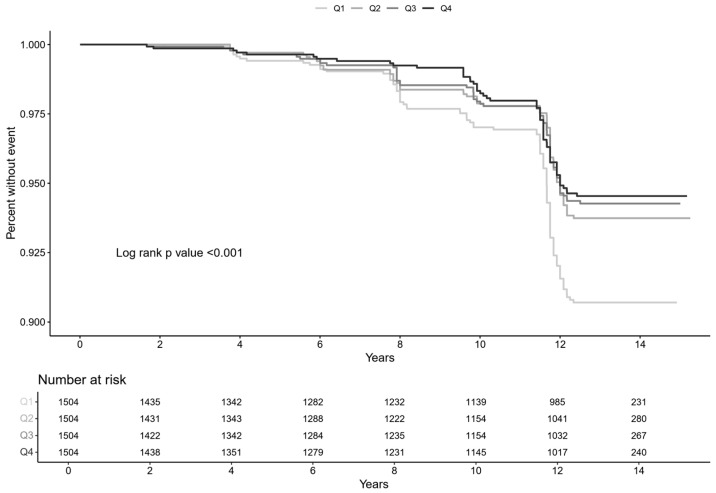
Kaplan–Meier curve for the development of incident muscle loss by ISI quartiles. The highest number of muscle loss events occurred in Q1, where insulin sensitivity was lowest, while the lowest number occurred in Q4, where insulin sensitivity was highest.

Over a mean follow-up duration of 11.5 ± 3.9 years, 311 subjects (5.2%) developed muscle loss (Table 2). The Kaplan–Meier plots demonstrated that the time to muscle loss development was significantly shorter in subjects within the lowest ISI quartile compared to those in the highest quartile (Figure 2). Cox proportional hazards model analyses showed a reverse-graded association between ISI and the risk of muscle loss. The hazard ratios (HRs) for the second, third, and highest ISI quartiles were 0.66 (95% confidence interval (CI), 0.49–0.89), 0.61 (95% CI, 0.45–0.82), and 0.58 (95% CI, 0.42–0.79), respectively, compared to the lowest quartile (Table 3).

**Figure 3:**
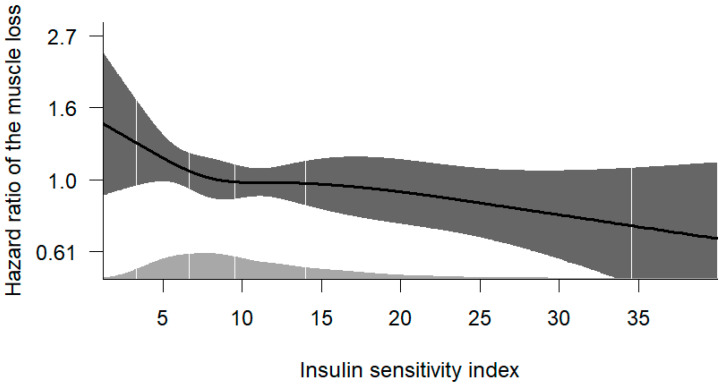
Restricted cubic spline curve for the development of incident muscle loss by ISI. Adjusted hazard ratios for muscle loss associated with the ISI were analyzed using a Cox model with restricted cubic splines. The dark gray zone represents confidence intervals, while the light gray zone with a mountain-like shape indicates population density along the spline variable. The model was adjusted for age, sex, BMI, SBP, eGFR, albuminuria, HDL-C, serum calcium, serum albumin, education, income, alcohol consumption, smoking, MET, and the presence of comorbidities such as cardiovascular disease (myocardial infarction, congestive heart failure, unstable angina, peripheral artery disease, and cerebrovascular disease), previous cancer, and COPD.

This association remained significant even after adjusting for potential confounders, including age, sex, BMI, SBP, HbA1c, eGFR, proteinuria, HDL-C, serum calcium, serum albumin, CRP and education level, economic status, alcohol consumption, smoking status, MET, history of cardiovascular disease (CVD), previous cancer history, and COPD history (Table 3). The HRs for these variables are provided in Supplemental Table S2. Spline analysis further revealed a negative correlation between the ISI score and the risk of muscle loss (Figure 3).

### 3.3. Association Between Insulin Sensitivity and All-Cause Mortality

**Figure 4:**
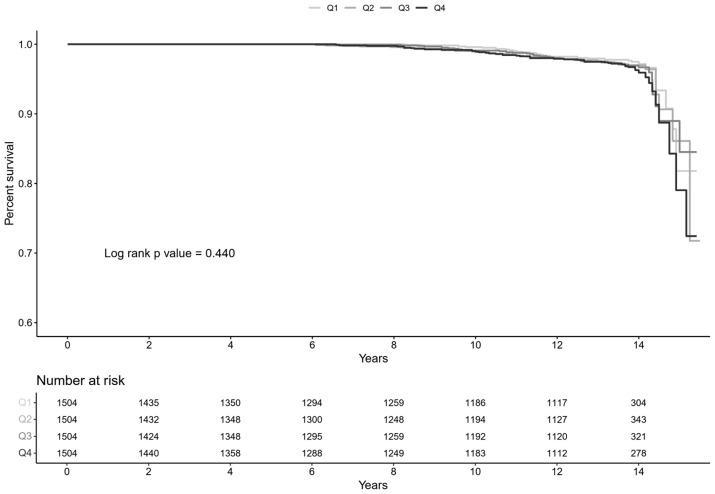
Kaplan–Meier curve for all-cause mortality by ISI quartiles. No statistically significant differences between the groups were observed.

A total of 168 (2.80%) all-cause mortality events were recorded during the follow-up period. No significant association was observed between insulin sensitivity and the risk of all-cause mortality (Figure 4). In a multivariable Cox regression analysis adjusted for demographic factors like age, sex, BMI, SBP, and education level, economic status, alcohol use, smoking status, MET, comorbidities (CVD, cancer, COPD), laboratory data (HbA1c, eGFR, proteinuria, serum calcium, serum albumin, CRP, and HDL-C), higher ISI quartiles were not significantly associated with a reduced risk of all-cause mortality compared to the lowest ISI quartile. The corresponding HRs with 95% CIs were 1.09 (0.69–1.72), 1.05 (0.66–1.68), and 1.13 (0.70–1.83), respectively (Table 3).

### 3.4. Risk of Muscle Loss with All-Cause Mortality

**Figure 5:**
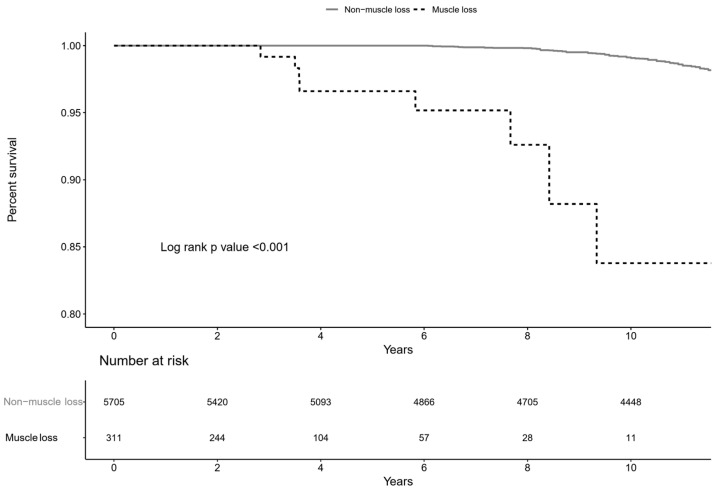
Kaplan–Meier curve for the development of all-cause mortality according to incident muscle loss. The curves showed that the muscle loss group (dashed line) had significantly higher mortality compared to the group without muscle loss (solid line).

To evaluate the prognostic significance of muscle loss, analyses were conducted to determine whether individuals with muscle loss had a heightened risk of mortality. All-cause mortality occurred in 8 (2.6%) subjects with muscle loss and 160 (2.8%) subjects without muscle loss (Table 4 and Figure 5). The multivariable Cox regression model, adjusted for confounding factors, showed that muscle loss was associated with a significantly higher risk of mortality, with a 15.9-fold increase (95% CI, 7.33–34.6).

## 4. Discussion

The relationship between insulin sensitivity and the risk of muscle loss was investigated in this long-term prospective observational cohort study. Findings revealed that enhanced insulin sensitivity was associated with a significantly lower risk of muscle loss compared to individuals with low insulin sensitivity. These results suggest that reduced insulin sensitivity in individuals without DM may contribute to muscle loss.

Insulin exerts anabolic effects on nitrogen-containing substrates, such as amino acids and proteins [29]. It plays a crucial role in preserving and restoring lean body mass and skeletal muscle. Furthermore, insulin is essential for supporting normal physiological growth during infancy and adolescence. It enhances glucose uptake by facilitating the translocation of glucose transporters to the cell membrane. Insulin also regulates key enzymes involved in glycolysis and gluconeogenesis and influences the expression of over 100 specific genes. Moreover, it promotes glucose uptake specifically in muscle tissue [30]. In muscle tissue, insulin promotes glucose uptake and facilitates protein synthesis by enhancing mRNA translation and increasing the availability of eIF4E through the regulation of 4E-BP1 [31]. Furthermore, insulin stimulates mRNA translation initiation by activating S6K1, which phosphorylates the ribosomal protein S6 [32]. Notably, the anabolic effects of insulin on skeletal muscle depend on adequate plasma amino acid concentrations [33].

Insulin sensitivity refers to the body’s responsiveness to insulin, particularly its ability to stimulate glucose uptake, promote peripheral glucose disposal, and suppress hepatic glucose production. Higher insulin sensitivity indicates that the body requires less insulin to effectively lower blood glucose levels. Conversely, reduced insulin sensitivity, known as insulin resistance, diminishes insulin’s effectiveness in muscle and adipose tissue, potentially resulting in a loss of lean body mass and fat mass.

Skeletal muscle plays a central role in insulin-mediated glucose uptake, accounting for approximately 60–70% of total insulin-stimulated glucose disposal in the body [34]. During euglycemic insulin clamp studies, over 80–90% of glucose disposal occurs in muscle tissue [19]. Consequently, muscle insulin resistance is a critical contributor to systemic insulin resistance. In conditions of insulin resistance, the ability of skeletal muscle to uptake glucose in response to insulin is markedly impaired.

Multiple studies have shown that muscle loss is common in patients with DM [9,11,12], with muscle mass declining more rapidly in individuals with DM compared to those without [11]. Mechanistically, several pathways contribute to muscle loss, including impaired insulin-stimulated glucose uptake, defects in insulin signaling, and various post-receptor intracellular abnormalities, such as reduced glucose transport, impaired glucose phosphorylation, decreased glucose oxidation, and reduced glycogen synthesis [12,35,36].

Skeletal muscle is the most significant insulin-dependent organ in the human body. Insulin suppresses the ATP–ubiquitin proteasome proteolytic pathway, a primary mechanism of muscle protein degradation [37]. The results of this study indicate that even in individuals without DM, lower insulin sensitivity is associated with an increased risk of muscle loss. This may occur because reduced insulin sensitivity diminishes the anabolic effects of insulin on skeletal muscle, ultimately leading to muscle loss.

The maintenance of skeletal muscle homeostasis depends on a delicate equilibrium between anabolic and catabolic processes [38]. At the molecular level, pathways such as PI3-K/Akt/mTOR, the ubiquitin–proteasome system, autophagy-dependent signaling, myostatin regulation, and inflammation play critical roles in preserving skeletal muscle mass [39]. This study is based on findings from a 16-year follow-up, during which age-related senescence may have also contributed to muscle loss.

Muscle loss is a natural part of aging [33,40,41,42]. However, excessive muscle loss can occur in individuals with both acute and chronic illnesses [5,11,30]. This loss of muscle strength and functional capacity contributes to an increased risk of mortality [9]. Muscle strength is inversely correlated with the risk of mortality [43], with low muscle strength and mass associated with dependency in daily activities [44] and an increased risk of falls, both of which can ultimately lead to mortality. Physical activity stimulates skeletal muscles to secrete myokines, which exert autocrine, paracrine, and endocrine effects. These endocrine functions of myokines play a crucial role in regulating body weight, enhancing insulin sensitivity, improving cognitive function, promoting bone healing, reducing inflammation, and inhibiting tumor growth by influencing adipose tissue, the liver, the gut, the brain, the pancreas, bone, immune cells, and tumors [45]. The loss of muscle mass can result in a decreased release of myokines, which play a vital role in maintaining overall health.

This study’s findings indicated that muscle loss was significantly associated with an increased risk of mortality. However, no notable correlation was observed between insulin sensitivity and the risk of all-cause mortality. This may be explained by this study’s focus on individuals without DM. As individuals progress from normal glucose tolerance to impaired glucose tolerance, insulin sensitivity decreases markedly, while the decline in glucose tolerance remains relatively modest due to a compensatory rise in insulin secretion [18]. Even in the presence of significant insulin resistance, functional β-cells can produce sufficient insulin to counteract impaired insulin action [19]. Complications of DM arise primarily from hyperglycemia. However, since this study was conducted on individuals without DM, it is likely that blood glucose levels remained within the normal range despite reduced insulin sensitivity, thus not influencing mortality. This study presents data from a 16-year follow-up period during which no association was found between insulin sensitivity and mortality. Nonetheless, longer follow-up periods might reveal different outcomes regarding the relationship between insulin sensitivity and mortality.

Insulin resistance, particularly in its early stages, can potentially be reversed through the adoption of a nutritious diet, regular physical activity, and the use of hypoglycemic medications [46]. This study’s results indicate that muscle loss increases mortality and that, in individuals without DM, lower insulin sensitivity is associated with an increased risk of muscle loss. Therefore, improving insulin sensitivity is important even in individuals without DM.

This study has several strengths, including its longitudinal design, the comprehensive assessment of muscle mass over a 16-year follow-up, detailed sociodemographic and comorbidity data, and rigorous adjustment for confounding variables. The BIA results revealed substantial differences in muscle loss across the four groups studied.

However, this study has some limitations. First, due to its observational nature, causal relationships between insulin sensitivity and muscle loss cannot be established. Also, follow-up research is needed to confirm this finding experimentally. Second, the KoGES did not include functional assessments such as muscle strength or physical performance, despite substantial evidence showing that these measures can predict clinically significant outcomes in older adults [47]. Incorporating functional evaluations would provide more meaningful insights into the clinical implications of muscle mass loss. Third, body composition was assessed exclusively using BIA. Although BIA is not the most accurate method for measuring muscle mass, as fat-free mass includes bodily fluids, bone, organs, and muscle, alternative methods such as DEXA, CT, or MRI offer more precise body composition measurements. Nonetheless, multifrequency BIA is a safe, cost-effective, and practical tool for assessing muscle and fat mass levels in clinical settings. Moreover, previous studies have demonstrated that the accuracy of BIA and DEXA measurements is comparable [48].

## 5. Conclusions

In conclusion, this long-term, prospective, community-based cohort study of middle-aged South Korean adults identified a reverse-graded relationship between insulin sensitivity and the risk of muscle loss, with the lowest risk observed in the highest ISI quartile. These findings suggest that higher insulin sensitivity may provide protective benefits. Furthermore, muscle loss was significantly associated with an increased risk of mortality. The results highlight the critical importance of early prevention of muscle loss and its significant public health implications.

## Figures and Tables

**Table 1:** Baseline characteristics of patients by ISI quartiles.

	ISI Quartiles				Total (*n* = 6016)
	Q1 (*n* = 1504)	Q2 (*n* = 1504)	Q3 (*n* = 1504)	Q4 (*n* = 1504)	
**Demographic data**					
Age (years)	51.1 ± 8.6	50.8 ± 8.5	50.6 ± 8.5	51.0 ± 8.8	50.9 ± 8.6
Sex (male%)	658 (43.8%)	652 (43.4%)	730 (48.5%)	809 (53.8%)	2849 (47.4%)
BMI (kg/m^2^)	25.9 ± 3.1	24.5 ± 2.9	23.9 ± 2.8	23.5 ± 2.9	24.5 ± 3.0
WHR	89.1 ± 7.5	87.0 ± 7.6	86.0 ± 7.4	85.7 ± 7.3	87.0 ± 7.6
SBP (mmHg)	123.3 ± 18.1	119.9 ± 18.2	117.4 ± 16.7	116.6 ± 16.8	119.3 ± 17.6
Economic status					
low	465 (30.9%)	467 (31.1%)	441 (29.3%)	477 (31.7%)	1850 (30.8%)
mid	417 (27.7%)	412 (27.4%)	477 (31.7%)	431 (28.7%)	1737 (28.9%)
high	622 (41.4%)	625 (41.6%)	586 (39.0%)	596 (39.6%)	2429 (40.4%)
Education level					
low	457 (30.4%)	423 (28.1%)	392 (26.1%)	425 (28.3%)	1697 (28.2%)
mid	815 (54.2%)	846 (56.3%)	886 (58.9%)	854 (56.8%)	3401 (56.5%)
high	232 (15.4%)	235 (15.6%)	226 (15.0%)	225 (15.0%)	918 (15.3%)
Smoking status					
Never	935 (62.2%)	943 (62.7%)	874 (58.1%)	830 (55.2%)	3582 (59.5%)
Former	265 (17.6%)	255 (17.0%)	256 (17.0%)	214 (14.2%)	990 (16.5%)
Current	304 (20.2%)	306 (20.3%)	374 (24.9%)	460 (30.6%)	1444 (24.0%)
Alcohol intake					
Never	741 (49.3%)	709 (47.1%)	681 (45.3%)	636 (42.3%)	2767 (46.0%)
Former	91 (6.1%)	86 (5.7%)	86 (5.7%)	85 (5.7%)	348 (5.8%)
Current	672 (44.7%)	709 (47.1%)	737 (49.0%)	783 (52.1%)	2901 (48.2%)
**Comorbidities**					
Hypertension	283 (18.8%)	189 (12.6%)	137 (9.1%)	116 (7.7%)	725 (12.1%)
Coronary artery disease	9 (0.6%)	10 (0.7%)	7 (0.5%)	7 (0.5%)	33 (0.5%)
Congestive heart failure	1 (0.1%)	1 (0.1%)	2 (0.1%)	3 (0.2%)	7 (0.1%)
Myocardial infarction	10 (0.7%)	8 (0.5%)	14 (0.9%)	6 (0.4%)	38 (0.6%)
Peripheral artery disease	4 (0.3%)	3 (0.2%)	4 (0.3%)	4 (0.3%)	15 (0.2%)
Cerebrovascular disease	15 (1.0%)	21 (1.4%)	16 (1.1%)	7 (0.5%)	59 (1.0%)
COPD	9 (0.6%)	8 (0.5%)	6 (0.4%)	10 (0.7%)	33 (0.5%)
Previous cancer history	33 (2.2%)	35 (2.3%)	37 (2.5%)	32 (2.1%)	137 (2.3%)
**Bioimpedance Analysis**					
LSM (kg)	44.3 ± 8.4	43.1 ± 7.9	43.3 ± 8.0	43.4 ± 7.8	43.5 ± 8.1
FFM (kg)	46.9 ± 8.8	45.7 ± 8.3	45.8 ± 8.4	46.0 ± 8.2	46.1 ± 8.4
FFMI (kg/m^2^)	18.2 ± 1.8	17.7 ± 1.8	17.7 ± 1.8	17.6 ± 1.8	17.8 ± 1.8
FM (kg)	19.6 ± 5.2	17.2 ± 5.0	15.8 ± 4.9	14.8 ± 5.1	16.9 ± 5.4
FMI (kg/m^2^)	7.8 ± 2.3	6.8 ± 2.2	6.2 ± 2.2	5.8 ± 2.3	6.7 ± 2.4
**MET**					
Q1 (<25th)	433 (28.8%)	397 (26.4%)	371 (24.7%)	368 (24.5%)	1569 (26.1%)
Q2 (25–49th)	419 (27.9%)	392 (26.1%)	381 (25.3%)	375 (24.9%)	1567 (26.0%)
Q3 (50–74th)	391 (26.0%)	438 (29.1%)	403 (26.8%)	389 (25.9%)	1621 (26.9%)
Q4 (≥75th)	261 (17.4%)	277 (18.4%)	349 (23.2%)	372 (24.7%)	1259 (20.9%)
**Laboratory parameters**					
eGFR (mL/min/1.73 m^2^)	90.6 ± 14.0	91.3 ± 14.3	93.3 ± 13.4	92.6 ± 13.5	91.9 ± 13.8
≥90	846 (56.3%)	884 (58.8%)	961 (63.9%)	916 (60.9%)	3607 (60.0%)
60–89	624 (41.5%)	588 (39.1%)	523 (34.8%)	566 (37.6%)	2301 (38.2%)
45–59	32 (2.1%)	28 (1.9%)	20 (1.3%)	20 (1.3%)	100 (1.7%)
<45	2 (0.1%)	4 (0.3%)	0 (0.0%)	2 (0.1%)	8 (0.1%)
Albuminuria (≥1+)	35 (2.3%)	35 (2.3%)	19 (1.3%)	20 (1.3%)	109 (1.8%)
BUN (mg/dL)	14.4 ± 3.6	14.1 ± 3.6	14.1 ± 3.5	14.4 ± 3.6	14.3 ± 3.6
Albumin (g/dL)	4.3 ± 0.3	4.3 ± 0.3	4.2 ± 0.3	4.3 ± 0.3	4.3 ± 0.3
CRP (mg/dL)	0.2 (0.1–0.3)	0.1 (0.1–0.2)	0.1 (0.1–0.2)	0.1 (0.0–0.2)	0.1 (0.1–0.2)
Calcium (mg/dL)	9.7 ± 0.4	9.6 ± 0.4	9.6 ± 0.5	9.6 ± 0.5	9.6 ± 0.5
Tchol (mg/dL)	196.6 ± 34.4	190.8 ± 34.1	187.1 ± 33.5	187.2 ± 35.2	190.4 ± 34.5
HDL-C (mg/dL)	42.6 ± 9.0	44.9 ± 10.1	45.8 ± 10.1	46.8 ± 10.4	45.0 ± 10.0
TG (mg/dL)	157.0 (115.0–216.0)	131.0 (98.0–181.0)	122.0 (92.0–168.0)	117.0 (89.0–158.0)	130.0 (96.0–181.0)
Fasting glucose (mg/dL)	86.1 ± 9.1	83.1 ± 8.1	81.5 ± 7.5	80.1 ± 8.1	82.7 ± 8.5
Hemoglobin (g/dL)	13.6 ± 1.6	13.5 ± 1.6	13.5 ± 1.6	13.6 ± 1.5	13.6 ± 1.6
HbA1c (%)	5.6 ± 0.4	5.5 ± 0.3	5.5 ± 0.3	5.5 ± 0.3	5.5 ± 0.3
HOMA-IR	2.4 ± 1.5	1.6 ± 0.5	1.3 ± 0.4	0.8 ± 0.4	1.5 ± 1.0
ISI	5.0 ± 1.2	8.1 ± 0.8	11.5 ± 1.3	23.2 ± 17.1	12.0 ± 11.0
IGI_60_	15.0 ± 56.2	9.3 ± 44.8	6.6 ± 54.0	3.8 ± 26.8	8.7 ± 47.1

Note: Data are presented as means ± standard deviations, frequencies (%), or as medians and interquartile ranges. Abbreviations: BMI, body mass index; BUN, blood urea nitrogen; COPD, chronic obstructive pulmonary disease; CRP, C-reactive protein; eGFR, estimated glomerular filtration rate; FFM, fat free mass; FFMI, fat free mass index; FM, fat mass; FMI, fat mass index; HDL-C, high-density lipoprotein–cholesterol; HOMA-IR, Homeostatic Model Assessment for Insulin Resistance; SBP, systolic blood pressure; HbA1c, glycated hemoglobin; IGI_60_, insulinogenic index; ISI, composite (Matsuda) insulin sensitivity index; LSM, lean soft mass; MET, metabolic equivalent of task; Tchol, total cholesterol; TG, triglyceride; WHR, waist–hip ratio.

**Table 2:** Muscle loss event rates among groups categorized by ISI quartiles.

	Total	ISI Quartiles			
		Q1	Q2	Q3	Q4
Number of participants	6016	1504	1504	1504	1504
Person–year	69,480	17,279	17,419	17,409	17,373
Muscle loss					
Events (%)	311 (5.2)	108 (7.2)	73 (4.9)	67 (4.5)	63 (4.2)
Events per 1000 person-yr	4.5	6.3	4.2	3.8	3.6
All-cause mortality					
Events (%)	168 (2.8)	36 (2.4)	41 (2.7)	4.3 (2.9)	48 (3.2)
Events per 1000 person-yr	2.4	2.0	2.3	2.4	2.7

Abbreviations: ISI, composite (Matsuda) insulin sensitivity index.

**Table 3:** Hazard ratios for the development of muscle loss by ISI quartiles.

	Model 1		Model 2		Model 3	
Groups	HR [95% CI]	*p*	HR [95% CI]	*p*	HR [95% CI]	*p*
Muscle loss						
Q1	1.00 [Reference]		1.00 [Reference]		1.00 [Reference]	
Q2	0.66 [0.49–0.89]	0.01	0.71 [0.52–0.97]	0.03	0.70 [0.51–0.94]	0.02
Q3	0.61 [0.45–0.82]	<0.01	0.73 [0.53–1.00]	0.05	0.69 [0.50–0.95]	0.02
Q4	0.58 [0.42–0.79]	<0.01	0.69 [0.49–0.98]	0.04	0.65 [0.46–0.92]	0.01
All-cause mortality						
Q1	1.00 [Reference]		1.00 [Reference]		1.00 [Reference]	
Q2	1.18 [0.75–1.85]	0.47	1.12 [0.71–1.77]	0.63	1.09 [0.69–1.72]	0.71
Q3	1.23 [0.79–1.92]	0.36	1.08 [0.68–1.72]	0.74	1.05 [0.66–1.68]	0.83
Q4	1.43 [0.93–2.21]	0.10	1.14 [0.71–1.84]	0.58	1.13 [0.70–1.83]	0.60

Note: Model 1: unadjusted model. Model 2: adjusted for age, sex, BMI, SBP, and laboratory parameters such as HbA1c, eGFR, proteinuria, HDL-C, serum calcium, serum albumin, and CRP. Model 3: adjusted for Model 2 plus education level, economic status, alcohol use, smoking status, MET, cardiovascular disease history, previous cancer history, and COPD history. Abbreviations: CI; confidence interval; BMI, body mass index; COPD, chronic obstructive pulmonary disease; CRP, C-reactive protein; eGFR, estimated glomerular filtration rate; HbA1c, glycated hemoglobin; HDL-C, high-density lipoprotein–cholesterol; HR, hazard ratio; ISI, composite (Matsuda) insulin sensitivity index; MET, metabolic equivalent of task; SBP, systolic blood pressure.

**Table 4:** Hazard ratios for all-cause mortality in individuals with muscle loss.

	Total	Non-Muscle Loss		Muscle Loss	
Number of participants	6016	5705		311	
Person–year	68,224	67,084		1141	
All-cause mortality					
Events (%)	168 (2.8)	160 (2.8)		8 (2.6)	
Events per 1000 person–year	2.5	2.4		7.0	
Cox proportional hazards model		HR [95% CI]	*p*	HR [95% CI]	*p*
Model 1		1.00 [Reference]		25.0 [11.8–53.0]	<0.01
Model 2		1.00 [Reference]		16.3 [7.52–35.1]	<0.01
Model 3		1.00 [Reference]		15.9 [7.33–34.6]	<0.01

Note: Model 1: unadjusted model; Model 2: adjusted for age, sex, BMI, SBP, and laboratory parameters such as HbA1c, eGFR, proteinuria, HDL-C, serum calcium, serum albumin, and CRP; Model 3: adjusted for Model 2 plus education level, economic status, alcohol use, smoking status, MET, cardiovascular disease history, previous cancer history, and COPD history. Abbreviations: CI; confidence interval; BMI, body mass index; COPD, chronic obstructive pulmonary disease; CRP, C-reactive protein; eGFR, estimated glomerular filtration rate; HbA1c, glycated hemoglobin; HDL-C, high-density lipoprotein–cholesterol; HR, hazard ratio; ISI, composite (Matsuda) insulin sensitivity index; MET, metabolic equivalent of task; SBP, systolic blood pressure.

## Data Availability

The data presented in this study are available on request from the corresponding author due to ethical issues.

## Supplementary Materials

The following supporting information can be downloaded at: https://www.mdpi.com/article/10.3390/jcm14041270/s1, Table S1. Diet intake information. Table S2. Hazards ratio of multivariate Cox regression for muscle loss.

## Abbreviations

The following abbreviations are used in this manuscript:

CI	Confidence interval
HOMA	Homeostasis model assessment
CRP	C-reactive protein
DEXA	Dual-energy X-ray absorptiometry
SBP	Systolic blood pressure
FFM	Fat-free mass
MET	Metabolic equivalent of task
CT	Computed tomography
FMI	Fat mass index
BIA	Bioelectrical impedance analysis
CVD	Cardiovascular disease
II	Insulin index
HDL	High-density lipoprotein
HDL-C	High-density lipoprotein cholesterol
BMI	Body mass index
ISI	Insulin sensitivity index
DM	Diabetes mellitus
HOMA-IR	homeostasis model assessment of insulin resistance
IDMS	Isotope dilution mass spectrometry
LSM	Lean soft mass
mRNA	messenger ribonucleic acid
eIF4E	eukaryotic initiation factor 4E
4E-BP1	4E-binding protein 1
S6K1	S6 kinase 1
ATP	adenosine triphosphate
