# Supplementary material for: Insulin Sensitivity and Muscle Loss in the Absence of Diabetes Mellitus: Findings from a Longitudinal Community-Based Cohort Study
Source: J Clin Med. 2025 Feb 14;14(4):1270. doi: 10.3390/jcm14041270 (PMC11856990; doi:10.3390/jcm14041270)
Supplement: Supplementary file 1 [file jcm-14-01270-s001.zip › jcm-3437738-supplementary.pdf]

Supplemental Table S1. Diet intake information

	Q1 *n* = 1,462	Q2 *n* = 1,467	Q3 *n* = 1,460	Q4 *n* = 1,463	p-value
**Energy (kcal)**	1858.3 (1575.5-2253.6)	1858.4 (1546.7-2227.0)	1847.1 (1525.5-2206.8)	1845.1 (1533.3-2209.3)	0.58
**Protein (g)**	63.4 (49.3-79.1)	63.2 (49.3-78.1)	61.5 (47.7-79.4)	61.9 (48.3-79.0)	0.60
**Fat (g)**	29.8 (20.0-41.8)	29.9 (20.0-40.6)	29.3 (19.5-41.7)	29.6 (20.0-41.6)	0.95
**Sugar (g)**	328.0 (283.7-387.5)	324.0 (278.3-382.9)	322.6 (279.5-376.1)	323.9 (277.6-375.7)	0.29
**Ca (mg)**	427.6 (300.3-594.3)	439.4 (308.7-603.8)	428.7 (292.7-617.0)	436.3 (302.7-606.4)	0.63
**P (mg)**	975.0 (771.7-1211.4)	973.3 (777.5-1212.7)	963.4 (745.0-1218.2)	967.0 (754.8-1213.6)	0.69
**Fe (mg)**	10.4 (7.8-13.3)	10.3 (7.8-13.3)	10.0 (7.3-13.3)	10.0 (7.5-13.0)	0.07
**K (mg)**	2396.8 (1814.5-3058.5)	2367.6 (1824.4-3036.0)	2337.9 (1703.1-3071.5)	2340.2 (1752.7-3017.9)	0.29
**Vit.A (R.E)**	444.9 (300.6-671.9)	455.9 (307.8-668.3)	438.1 (290.0-645.0)	439.3 (293.7-637.8)	0.19
**Na (mg)**	2964.1 (2101.0-3980.1)	2957.8 (2140.7-3909.9)	2894.7 (2109.7-3964.7)	2930.5 (2139.5-3854.5)	0.93
**Vit.B1 (mg)**	1.2 (0.9-1.5)	1.2 (0.9-1.5)	1.2 (0.9-1.5)	1.2 (0.9-1.5)	0.31
**Vit.B2 (mg)**	1.0 (0.7-1.2)	0.9 (0.7-1.2)	0.9 (0.7-1.2)	0.9 (0.7-1.2)	0.74
**Niacin (mg)**	14.9 (11.6-18.9)	14.8 (11.5-18.5)	14.6 (11.3-18.8)	14.7 (11.4-18.6)	0.64
**Vit.C (mg)**	106.1 (73.3-157.7)	105.1 (70.9-152.9)	100.4 (67.6-152.9)	99.7 (67.8-151.8)	0.06
**Zinc (μg)**	8.1 (6.4-10.3)	8.1 (6.4-10.2)	7.9 (6.2-10.2)	8.0 (6.4-10.0)	0.27
**Vit.B6 (mg)**	1.7 (1.3-2.1)	1.7 (1.3-2.1)	1.6 (1.3-2.1)	1.7 (1.3-2.1)	0.24

**Folate (μg)**	225.1 (170.2-300.9)	227.6 (171.3-298.3)	220.0 (161.6-298.9)	221.4 (164.7-289.1)	0.06
**Retinol (μg)**	56.9 (29.1-92.8)	57.9 (27.6-93.5)	56.0 (28.1-96.1)	61.6 (29.4-95.5)	0.54
**Carotene (μg)**	2159.2 (1435.9-3348.6)	2166.2 (1459.0-3400.1)	2054.2 (1368.2-3242.7)	2112.7 (1386.8-3162.2)	0.13
**Ash (mg)**	17.2 (12.6-24.4)	16.8 (12.6-23.8)	16.7 (12.2-24.7)	16.8 (12.5-24.1)	0.67
**Fiber (g)**	6.6 (4.8-8.5)	6.5 (4.9-8.4)	6.4 (4.6-8.6)	6.3 (4.7-8.2)	0.06
**Vit.E (mg)**	8.6 (6.2-12.0)	8.5 (6.3-11.5)	8.3 (5.9-11.7)	8.4 (5.9-11.4)	0.09

Supplemental Table S2. Hazards ratio of multivariate Cox regression for muscle loss

Covariate		Hazard ratio	95% CI	p-value
**Age**		1.110	1.095-1.125	<0.001
**Sex**	**Male**	Reference		
	**Female**	2.747	2.126-3.550	<0.001
**BMI**		1.113	1.074-1.153	<0.001
**SBP**		1.026	1.020-1.031	<0.001
**HbA1c**		2.236	1.603-3.118	<0.001
**Proteinuria**		0.735	0.490-1.100	0.135
**eGFR**		0.972	0.965-0.979	<0.001
**Albumin**		0.501	0.346-0.726	<0.001
**Calcium**		0.445	0.349-0.568	<0.001
**CRP**		1.185	0.984-1.428	0.074
**HDL-C**		0.997	0.986-1.008	0.602
**Education level**	**Low**	Reference		
	**Mid**	0.312	0.247-0.393	<0.001
	**High**	0.177	0.112-0.285	<0.001
**Economic status**	**Low**	Reference		

	**Mid**	0.402	0.308-0.525	<0.001
	**High**	0.179	0.131-0.245	<0.001
**Alcohol intake**	**Never**	Reference		
	**Former**	0.758	0.468-1.223	0.262
	**Current**	0.471	0.369-0.600	<0.001
**Smoking status**	**Never**	Reference		
	**Former**	0.467	0.323-0.675	<0.001
	**Current**	0.373	0.261-0.531	<0.001
**MET**		1.000	1.000-1.000	<0.001
**CVD history**		1.886	1.059-3.358	0.031
**COPD history**		1.322	0.329-5.310	0.694
**Cancer history**		1.578	0.864-2.879	0.138

Abbreviations: BMI, body mass index; COPD, chronic obstructive pulmonary disease; CVD, cardiovascular disease; CRP, C-reactive protein; eGFR, estimated glomerular filtration rate; HDL-C, high density lipoprotein-cholesterol; SBP, systolic blood pressure; HbA1c, glycated hemoglobin; MET, metabolic equivalent of task.
